# Two-Dimensional Transition Metal Carbides and Nitrides (MXenes) for Water Purification and Antibacterial Applications

**DOI:** 10.3390/membranes11110869

**Published:** 2021-11-12

**Authors:** Inamullah Mahar, Fida Hussain Memon, Jae-Wook Lee, Kyung Hwan Kim, Rafique Ahmed, Faheeda Soomro, Faisal Rehman, Ayaz Ali Memon, Khalid Hussain Thebo, Kyung Hyun Choi

**Affiliations:** 1 National Centre of Excellence in Analytical Chemistry (NCEAC), University of Sindh, Jamshoro 76060, Sindh, Pakistan; inamullah@gmail.ac.kr (I.M.); ayazmemon33@usindh.edu.pk (A.A.M.); 2 Department of Electrical Engineering, Sukkur IBA University, Sukkur 65200, Sindh, Pakistan; fida.hussain@iba-suk.edu.pk; 3 Advanced Micro Mechatronics Lab., Department of Mechatronics Engineering, Jeju National University, Jeju-si 63243, Korea; jaewook482@gmail.com (J.-W.L.); kyunghwankim@jejunu.ac.kr (K.H.K.); 4 Institute of Composite Science Innovation (InCSI), School of Materials Science and Engineering, Zhejiang University, 38 Zheda Road, Hangzhou 310027, China; Rafiqueahmed116@hotmail.com; 5 Department of Linguistics and Human Sciences, Begum Nusrat Bhutto Women University, Sukkur 65200, Sindh, Pakistan; faheedasoomro@gmail.com; 6 Department of Mechatronics Engineering, College of EME, National University of Sciences and Technology (NUST), Peshawar Road, Rawalpindi 43701, Punjab, Pakistan; Faisal.rehman.be13@iba-suk.edu.pk; 7 Institute of Metal Research, Chinese Academy of Sciences (CAS), Shenyang 110016, China

**Keywords:** two-dimensional, MXenes, membrane, water purification, antibacterial

## Abstract

Two-dimensional (2D) materials such as graphene, graphene oxide (GO), metal carbides and nitrides (MXenes), transition metal dichalcogenides (TMDS), boron nitride (BN), and layered double hydroxide (LDH) metal–organic frameworks (MOFs) have been widely investigated as potential candidates in various separation applications because of their high mechanical strength, large surface area, ideal chemical and thermal stability, simplicity, ease of functionalization, environmental comparability, and good antibacterial performance. Recently, MXene as a new member of the 2D polymer family has attracted significant attention in water purification, desalination, gas separation, antibacterial, and antifouling applications. Herein, we review the most recent progress in the fabrication, preparation, and modification methods of MXene-based lamellar membranes with the emphasis on applications for water purification and desalination. Moreover, the antibacterial properties of MXene-based membranes show a significant potential for commercial use in water purification. Thus, this review provides a directional guide for future development in this emerging technology.

## 1. Introduction

The modern world is facing a huge crisis for clean drinking water. Specifically, the populations of underdeveloped and developing countries have been exposed to multiple waterborne fatal diseases, waterborne epidemics, and waterborne insect-based outbreaks [1,2,3,4,5,6]. Water purification and desalination are considered to be the biggest challenges of this century and various technologies have been developed to fulfil increasing water demands for drinking and agriculture around the world since the 1950s [7,8,9]. Most were unsuccessful due to high costs, environmental incomparability, a low efficiency and stability, and several fouling issues. Recently, membrane technology has been proven to be versatile for water purification, desalination, and industrial wastewater applications and has significantly improved the quality of life on the planet [10]. Membrane technology possesses a number of advantages such as a reasonable cost, environmental compactness, energy saving, simplicity, and good selectivity compared with traditional separation methods including adsorption, absorption, filtration, and distillation [11,12,13].

Currently, a number of materials such as carbon nanotubes (CNTs), MOFs, COFs, zeolite, polymers, ceramics, graphene, GO, and boron nitride [14,15,16,17,18,19,20,21,22,23,24,25,26,27,28] have been widely explored in various water purification separation processes. Among them, polymers are commercially used in materials, biomaterials, and membrane technology due to their high packing density, biocompatibility, low cost, and large-scale application in organic and biomedical engineering [29,30,31,32,33,34,35,36]. However, issues such as swelling in organic solvents, fouling in an aqueous medium, and a low stability at high temperatures limit their application [37]. There is a dire need of an alternative polymer material that overcomes the aforementioned limitations.

The recent emergence of 2D MXenes has shown their potential as an ideal material for separation applications owing to their outstanding stability in water, impressive strength, single atomic thickness, good antibacterial properties, high adsorption, and molecular filtration abilities [38,39,40,41,42,43,44,45,46,47,48,49,50,51,52,53,54,55,56,57,58,59,60,61,62]. The common formula of MXene is Mn_+1_X_n_T_x_ (n = 1–3), where M is the transition metal (i.e., V, Mo, Cr, Hf, Zr, Ta, Ti), X represents carbon and nitrogen atoms, and Tx stands for the terminated OH, O, and F atoms. Due to these surface termination groups, MXene is easily dispersed in water as well as in several organic solvents making it an ideal material for various separation applications in water purification, desalination, organic separation nanofiltration, and biomedical applications. Despite these advantages, only a few MXene members have been explored as separation membranes for water purification and antibacterial studies.

It is necessary to evaluate the current development of MXene-based lamellar membranes for antibacterial and separation utilization. In this short review, the current fabrication and development methods of MXene-based membranes are summarized. MXene-based membranes for desalination, water refinement, and antibacterial applications are discussed in detail. Lastly, this review concludes with several future prospects and recommendations to improve the physicochemical properties of membranes.

## 2. Fabrication of MXene-Based Membranes

**Figure 1:**
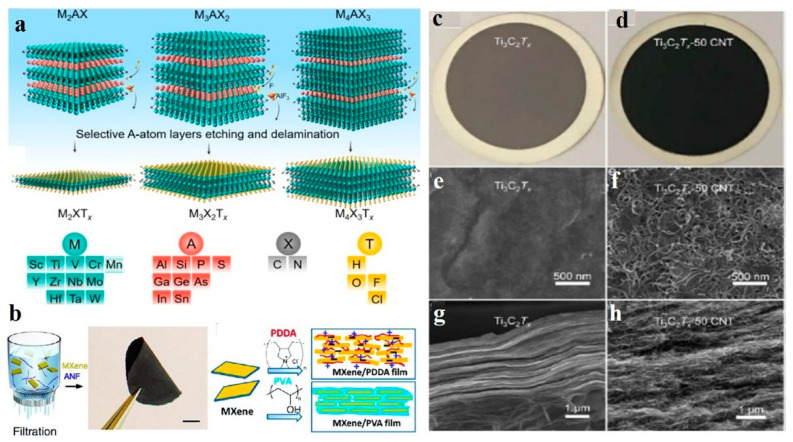
(**a**) MXene precursors and their common synthesis methods. Reprinted with permission from [70]. Copyright 2020 Springer Nature Group. (**b**) Fabrication of MXene/polymer-based composite membrane by the VF method. Reprinted with permission from [63]. Copyright 2015 American Chemical Society. (**c**,**d**) Fabrication of pristine Ti_3_C_2_Tx and Ti_3_C_2_Tx-CNT composite membranes. (**e**) The digital photograph of the solutions. (**f**) AFM study of Ti_3_C_2_Tx nanosheets. (**g**,**h**) Digital photos: surface; cross-sectional SEM images of pristine Ti_3_C_2_Tx, Ti_3_C_2_Tx-CNT, and CNT membranes, respectively. Reprinted with permission from [68]. Copyright 2020 American Chemical Society.

MXene (Figure 1a) is a younger member of the 2D family and has been widely fabricated into both laminar as well as pristine nanosheet membranes using different methods. The ideal filtration membranes should be defect free, ultrathin, a dense film and mechanically robust with a high selectivity for small molecules along with good antifouling and antibacterial properties. Generally, methods such as spin coating, spray coating, vacuum filtration, Langmuir–Blodgett, drop casting, or direction evaporation and dip coating are used for the fabrication of 2D MXene lamellar membranes. Gogotsi et al. [63] used a vacuum filtration method for first time to prepare freestanding and PVDF-supported 2D Ti_3_C_2_T_x_-based membranes. Such membranes demonstrated good hydrophilic properties because of the presence of the useful group in conjunction with excellent elasticity as well as a good mechanical strength, which is an ideal potential in separation membranes. Among these methods, the VF method is widely used for the fabrication of 2D MXene-based membranes due to its simplicity and ease of operation (Figure 1b). Ding et al. [64] also reported 2D MXene (Ti_3_C_2_T_x_) with enhanced properties using a vacuum filtration method on a porous support whereas Kang et al. [65] fabricated MXene (Ti_3_C_2_T_x_) and GO-based composite membranes by the same method. Sun and coworkers also fabricated GO/MXene lamellar membranes by the filtration method [66]. Wang and co-researchers worked out an improvement of the microstructure and physiochemical properties of an MXene membrane by mixing it with a polymer matrix. Recently, Wang et al. [67] reported Ti_3_C_2_T_x_ lamellar membranes produced by employing a multivalent ion as a hydrogel pillar in the interlayer spacing. Researchers for obtaining the uniform composition mixed a solution of sodium alginate (SA) and MXene Ti_3_C_2_T_x_; this composite, SA-Ti_3_C_2_T_x_, was then used for the lamellar SA-Ti_3_C_2_T_x_ membranes. Fascinatingly, a molecule of SA attached onto the MXene sheets by hydrogen bonding and Van der Waals forces. Finally, pillared SA-Ti_3_C_2_T_x_ laminates were arranged by submerging an SA-Ti_3_C_2_Tx membrane into a solution of different types of multivalent cations such as Ca^2+^, Ba^2+^, Mn^2+^, and Al^3+^. The pillar membrane showed a homogeneous structure similar to a nacre-like composite and it considerably decreased the swelling effect. Liu et al. [68] fabricated Ti_3_C_2_Tx-CNT hybrid membranes using vacuum filtration (Figure 1c,d). Liu and coworkers also fabricated pristine Ti_3_C_2_Tx and CNT membranes for comparative studies using the VF method (Figure 1g,h). They improved the mechanical stability and permeance of MXene by incorporating CNTs into Ti_3_C_2_Tx nanosheets. Huang et al. [48] used a phase inversion process to fabricate a PES-Ni@MXene membrane by using an external field and incorporated magnetic Ni@MXene nanoparticles with the upper layer of the PES membrane during a wet phase inversion process. MXene-based lamellar membranes were also prepared by a layer-by-layer (LbL) method [69]. Tian et al. assembled a tris(2-aminoethyl) amine (TAEA) molecule and Ti_3_C_2_Tx MXene using an LbL assembly and obtained highly ordered multilayer of MXene/TAEA with an interlayer distance ~1 Å. This strategy was a good addition to fabricate MXene-based multilayered membranes for large-scale applications.

From the above studies, it was concluded that the vacuum filtration method was mostly used to fabricate MXene membranes. However, there are several disadvantages associated with the vacuum filtration method. It needs a large volume of solvent, takes long time, and is definitely difficult to scale up. Therefore, alternative methods such as the shear alignment method, printing method, and spin coating method should be utilized to fabricate state-of-the-art MXene-based laminar membranes with advanced physicochemical properties to fully utilize the power of this wonder material.

## 3. Water Purification and Desalination Applications

**Figure 2:**
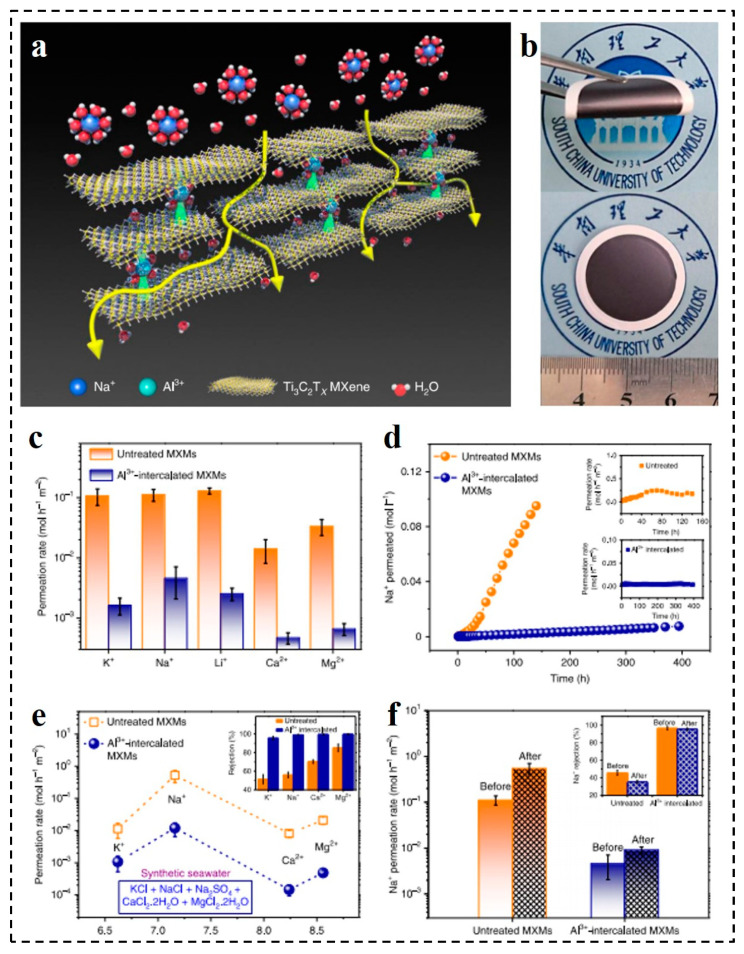
Ti_3_C_2_Tx MXene membranes (MXM): (**a**) Schematic representation of Al^3+^ intercalation between two adjacent MXene layers and thus a fixed d-spacing. The hydrated cation such as Na+ are rejected and the water molecules can permeate through the MXM. (**b**) Digital photos of untreated MXM and its blending state. (**c**–**f**) The ion permeation rate and DI permeance (**c**); time-dependent Na^+^ permeation (**d**); comparison of the rates of ions in synthetic seawater (**e**); chlorine resistance (**f**); of untreated MXMs and Al^3+^-intercalated MXMs. Reprinted with permission from [86]. Copyright 2020 Nature Publishing Group.

Water pollution and desalination are the most important issues in recent days, which greatly affect the quality of life on earth. The commercially used membranes for water purification and desalination are suppressed by antifouling problems that decrease the life of the membrane and increase the operational cost. Therefore, researchers focus on introducing novel materials to enhance the antifouling property of the membrane to save energy and the operational cost. Recently, MXene-based membranes have been widely used for wastewater treatment, water purification, and desalination applications, as shown in Table 1 [71,72,73,74,75,76,77,78,79,80,81,82]. Initial work by Gogotsi et al. [63] demonstrated 2D MXene-based membranes with PVDF supported particular ion separations; for instance, Mg^2+^, Ca^2+^, Li^+^, Al^3+^, Ni^2+^, Na^+^, and K^+^. The fabricated membrane exhibited a good permeability of ~37.4 L m^−2^h^−1^ bar^−1^. Metal ions having lesser hydration radii and a larger charge than the space between the MXene interlayers (~6 A°) indicated a slow permeability compared with single-charged cations. Such membranes have been inspected for antibacterial and biofouling properties of a single and a few layers of Ti_3_C_2_T_x_ MXene flakes in a colloidal solution. Liu et al. [83] produced a Ti_3_C_2_T_x_ membrane on polyacrylonitrile for NaCl salt separation. A 60 nm-thick membrane exhibited 99.5% separation efficiency and water permeance up to ~85 L m^−2^ h^−1^ bar^−1^ at 65 °C. Mahmoud et al. [84] boosted the antifouling properties, water permeance, and rejection through an MXene/Ag composite; the fabricated composite was 470 nm of thickness with 21% loading of AgNPs. Compared with pristine MXene laminates (~118 L m^−2^ h^−1^ bar^−1^), the prepared composite membrane showed ~420 L m^−2^ h^−1^ bar^−1^ water permeance under the same condition. This membrane also proved to have the highest stability in synthetic sea water. Lu et al. [85] validated a self-crosslinked MXene membrane (SCMMs) for the monovalent separation of ions. The terminal functional groups of MXene nanosheets were crosslinked with each other through a self-crosslinking reaction (–H + –OH = –O– + H_2_O). By this approach, the swelling of MXene decreased up to 15.4 Å compared with a pristine MXene membrane (16.6 Å) and showed outstanding constancy for 70 h. The permeation rate of SCMMs was near to two orders of magnitude less than the pristine MXene membrane, which specified the noticeably better enactment of the ion segregation by self-crosslinking between the MXene nanosheets. These membranes also indicated comparatively few monovalent ion permeation rates where the rates of permeation for Li^+^ (hydrated diameter of 7.65 Å), Na^+^ (hydrated diameter of 7.16 Å), and K^+^ (hydrated diameter 6.62 Å) were 0.0283, 0.222, and 0.232 mol h^−1^ m^−2^, respectively. Similar to other 2D materials, MXene also has a water swelling property and delaminates easily because of hydrogen bonding and electrostatics, that affect the separation efficiency of the membranes. Wang et al. [67] arranged pillared lamellar membranes (M-SAT) with improved separation properties. Metal cations such as Mn^2+^ and Ca^2+^ enlarged the d-spacing from 13.8 to 15.2 Å of a pristine Ti_3_C_2_T_x_ membrane. These metal cations were presented as a support between the MXene nanosheets. An improvement in the selectivity of the 2D laminates highly depended upon the d-spacing. An ultrathin M-SAT pillared membrane expressed 100% rejection of Na_2_SO_4_ salt with an improvement in the d-spacing. As associated to the earlier reported MXene-based membrane, this showed less swelling. Ding et al. [86] developed non-swelling MXene laminates for water cleansing with enriched separation properties (Figure 2a,b) by inserting Al^3+^ ions between MXene nanosheets (Figure 2a). These ions produced a strong electrostatic interaction with the functional groups of MXene. In the end, the membranes exhibited less swelling in the water and the stability was increased for up to 400 h. Moreover, these laminates presented 89.5–99.5% rejection against NaCl with better water permeance of 1.1–8.5 Lm^−2^ h^−1^ bar^1^, as shown in Figure 2c–f.

**Figure 3:**
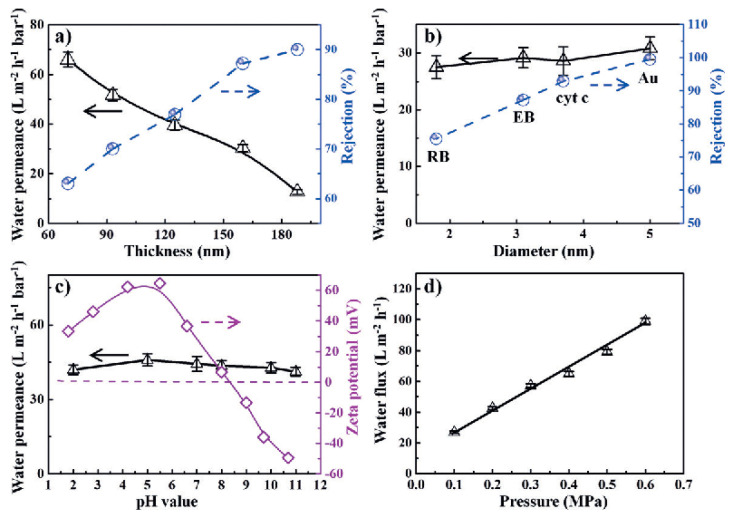
MXene-based (g-C_3_N_4_) nanosheet membranes for dye separation: g-C_3_N_4_ nanosheet membranes; separation performance of g-C_3_N_4_ membranes against different dye molecules. (**a**) Separation performance based on the thickness of the membranes; (**b**) against the diameter (size) of each probe molecule; (**c**) water permeance and zeta potential of the membrane in different pH environments; (**d**) water permeability based on variable pressure. Reprinted with permission from [87]. Copyright 2020 American Chemical Society.

As for molecular separation, Wang et al. [87] demonstrated a novel type of 2D g-C_3_N_4_ nanosheet membrane with artificial nanopores for water purification. These nanosheet membranes were exploited for the removal of distinct types of molecules, for example, Evan blue (EB), rhodamine B (RB), gold nanoparticles (Au NPs), and cytochrome C (Cytc), as shown in Figure 3a,b. The rejection percentages of 87.2%, 75.5%, 99.5%, and 93.1% for EB, RB, Au NPs, and Cytc were obtained, respectively. The 160 nm-thick membrane showed water permeance up to ~29 L m^−2^ h^−1^ bar^−1^. Additionally, the permeability of the membranes against variable thicknesses and pressures was also validated (Figure 3c,d).

**Figure 4:**
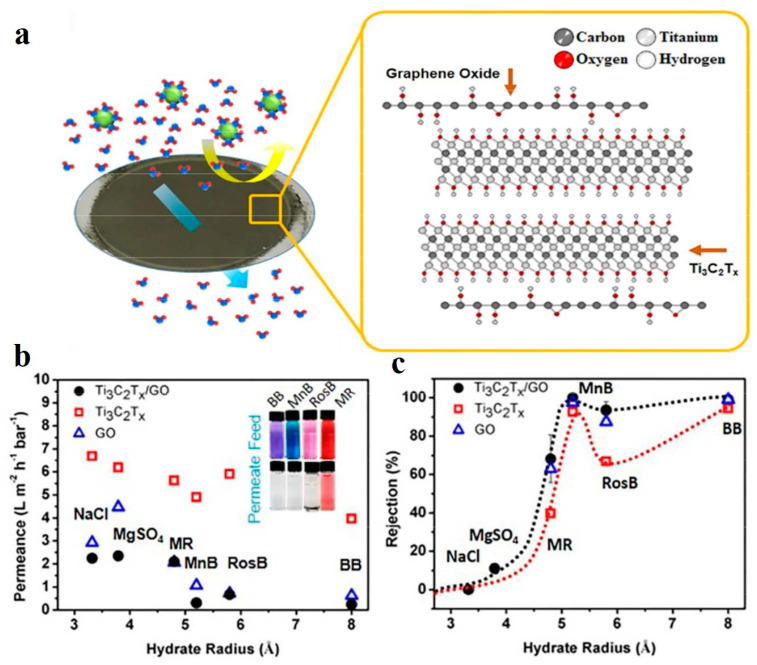
(**a**) Digital photograph of an MXene (Ti_3_C_2_T_X_)/GO-based composite membrane for water purification and a schematic representation of an ionic and molecular sieving mechanism. (**b**) Separation efficiency of various probe molecules with different sizes through a 90 nm-thick Ti_3_C_2_T_x_/GO composite membrane before and after filtration. (**c**) The separation performance of a pristine GO, MXene, and MXene/GO composite membrane against different probe molecules. Reprinted with permission from [65]. Copyright 2017 American Chemical Society.

Han et al. [88] demonstrated PES/MXene ultrafiltration membranes for the separation of Congo red (CR) dye. The as-prepared membrane showed pure water permeance of ~115 Lm^−2^ h^−1^ and a 92.3% rejection of the CR dye at 0.1 MPa. Kang and coworkers fabricated a Ti_3_C_2_Tx/GO composite membrane with a thickness of 90 nm, as shown in Figure 4a [65]. The interlayer of the as-prepared membrane was around 5 Å due to swelling in the water. Such membranes showed > 99% for those molecules, which had radii greater than 5 Å at a pressure of 5 bar and obtained 68%, 99.5%, 93.5%, and 100% rejection of methyl blue (MB, hydrated radii 4.87), methylene blue (MLB, 5.04), rose Bengal (RB, 5.88), and brilliant blue (BB, 7.98), respectively (Figure 4b,c). Recently, Liu et al. [66] also fabricated an MXene/GO membrane and used it for the separation of small organic dyes such as NR, MB, CV, and BB. The membrane showed a >99% rejection of these dyes. Similar membranes also showed an excellent rejection of bovine serum albumin (BSA) and humic acid (HA) molecules. However, these membrane showed less permeability compared with previously reported MXene/GO membranes [65]. Various studies have shown that pH, temperature, pressure, and water uptake significantly affect the microstructural properties and separation performance of 2D membranes. Ding and coworkers used a VF technique to develop a 2D Ti_3_C_2_T_x_-based novel membrane on an AAO support [64]. The as-prepared membranes showed outstanding water permeability for DI water up to ~1000 Lm^−2^ h^−1^ bar^1^. Furthermore, the separation effectiveness of laminates with different sizes of molecules was estimated by Ding et al. [64]. For molecules having a diameter larger than 2.5 nm, the membrane showed more than a 90% rejection rate. Ma et al. [89] fabricated a P84-copolyimide/MXene-based membrane with an excellent water permeability of ~268 L m^−2^ h^−1^ bar^−1^ at 0.1 MPa and ensured a 99% rejection of gentian violet dye. Shen et al. [77] reported MXene/PSF (Ti_3_C_2_Tx) membranes with a 99% rejection of a BSA molecule and water permeance up to ~218 Lm^−2^ h^−1^ bar^−1^. Recently, Liu and coworkers demonstrated a Ti_3_C_2_Tx/CNT hybrid membrane for the recovery of precious metals, i.e., gold (Au) [68]. The as-prepared membranes showed an excellent ability to capture Au (III) up to 99.8% and the water permeance reached up to ~437.6 Lm^−2^ h ^−1^ bar^−1^, approximately 202 times higher than the pristine MXene membrane reported.

Many works have described MXene-based lamellar membranes for desalination and water purification. Most of the MXene laminates were arranged on a polymeric substrate for superior performance and rejection. MXenes also have a weak bond with substrates; the resulting membranes can be easily destroyed and this greatly affects the separation performance of the membranes. As with GO laminates, MXene has also showed a good water permeance due to its hydrophilic nature. MXene-based membranes allowed cations with a larger hydration radii than the interlayer space between the MXene interlayers (0.6 nm). Furthermore, if the cations showed a greater charge, the membrane performed a slow permeation compared with the single-charged cations. Hence, the physicochemical and separation properties of MXene-based membranes can be improved by using suitable intercalation or crosslinking to control the pore structure. Further 2D–2D intercalations can also improve the permeance and selectivity of the membranes.

## 4. Antibacterial Activity of MXene-Based Membranes

**Figure 5:**
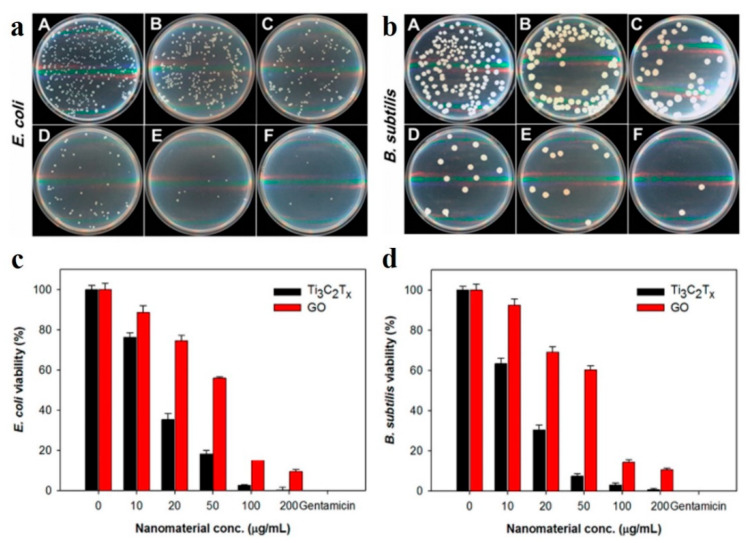
Ti_3_C_2_T_x_ nanosheet membranes. (**a**) Antibacterial activities of Ti_3_C_2_T_x_ membranes in an aqueous solution against *E. coli* and (**b**) *B. subtilis* with different concentrations, i.e., 0 µg/mL (**A**), 10 µg/mL (**B**), 20 µg/mL (**C**), 50 µg/mL (**D**), 100 µg/mL (**E**), and 200 µg/mL (**F**), respectively. (**c**,**d**) Cell viability measurement and comparison studies of Ti_3_C_2_T_x_ and GO membranes against *E. coli* and *B. subtilis* bacterial strains. Bacterial suspensions (107 CFU/mL) were incubated with different concentrations (0–200 µg/mL) of Ti_3_C_2_T_x_ and GO membranes at 35 °C for 4 h at a speed of 150 rmp. Reprinted with permission from [97]. Copyright 2016 American Chemical Society.

Pathogenic contamination is considered to be the most harmful issue worldwide and is responsible for various kinds of waterborne diseases [90]. It is directly responsible for the biofouling of any water filtration membrane; therefore, it is important that a membrane should be tested against antibacterial properties. Up to date, several bactericidal nanomaterials including graphene, TMDCs, and MXenes have been explored to meet these challenges. The antibacterial activity of graphite, graphite oxide, GO, rGO, MoS_2_, and WS_2_ against Gram-negative and Gram-positive bacteria have already been tested. Recently, MXenes with unique hydrophilic properties, a good adsorption, an ideal surface functionality, and excellent biocompatibility and photothermal properties have been widely tested for wastewater treatment and desalination, water purification, ion separation and other applications, as shown in Table 1. MXenes are expected to be resistant to biofouling and offer bactericidal properties [91]. However, very few studies [91,92,93,94,95,96,97,98,99] have been carried out in this direction. An initial work by Rasool et al. [97] reported that Ti_3_C_2_Tx membranes could be an ideal platform for antibacterial studies (Figure 5a–d). Rasool et al. [97] further used Ti_3_C_2_T_x_-based membranes to measure the antibacterial properties against *Escherichia coli* (*E. coli*) and *Bacillus subtilis (B. subtilis*) by using bacterial growth curves based on optical densities (OD) and colony growth on agar nutritive plates (Figure 5b,c). The membranes showed a high antibacterial efficiency against both Gram-negative *E. coli* and Gram-positive *B. subtilis* compared with the GO membranes. Concentration-dependent antibacterial activity was observed and more than 98% of bacterial cell viability loss was found at 200 μg/mL in Ti_3_C_2_T_x_ for both bacterial cells within 4 h of exposure, as confirmed by a colony-forming unit (CFU) and regrowth curve (Figure 5d,e). In another study, Ti_3_C_2_T_x_/PVDF composite membranes were tested to measure the antibacterial rate of *E. coli* and *B. subtilis* [91]. The composite membranes showed a ~73% and ~63% antibacterial rate for *B. subtilis* and *E. coli*, respectively, compared with the control PVDF membranes [91]. Additionally, the Ti_3_C_2_T_x_ membrane showed over a 99% growth inhibition of both bacteria under the same conditions. Mayerberger et al. [92] demonstrated Ti_3_C_2_Tz/chitosan composite nanofiber membranes for a passive antibacterial wound dressing application. The as-prepared composite membrane showed a 95% and 62% reduction in the colony-forming units of Gram-negative *E. coli* and Gram-positive *Staphylococcus aureus* (*S. aureus*), respectively. Jastrzebsa and coworkers also reported the antimicrobial properties of a Ti_3_C_2_ MXene-based nanocomposite, i.e., Ti_3_C_2_/SiO_2_/Ag, Ti_3_C_2_/Al_2_O_3_/Ag, and Ti_3_C_2_/SiO_2_/Pd [93]. They also demonstrated the outstanding bioactive properties of Ti_2_C and Ti_3_C_2_ MXenes against a Gram-negative bacterial strain [99]. Recently, Zhu et al. [95] evaluated the effect of near-infrared (NIR) light on the antibacterial activities of silver (Ag), Ti_3_C_2_T*_x_*, and an Ag/Ti_3_C_2_T*_x_* composite. The as-prepared Ag/Ti_3_C_2_T*_x_* composite showed a high efficacy against Gram-positive *S. aureus* and Gram-negative *E. coli* bacteria in an in vitro antibacterial test. Upon NIR irradiation, the antimicrobial effect of Ag/Ti_3_C_2_T*_x_* significantly strengthened compared with the pristine Ag and Ti_3_C_2_T_x_. The growth of *E. coli* was completely inhibited during the initial 0–6 h by 200 µg/mL of Ti_3_C_2_T_x_ due to the photothermal heat produced killing the bacteria in the surrounding area. The Ag/Ti_3_C_2_T_x_ composite exhibited the best antibacterial activities with the same dose of pristine Ag and Ti_3_C_2_T_x_. After NIR irradiation, the Ti_3_C_2_T_x_ composite could completely restrain the *E. coli* growth when used at 100–200 µg/mL.

## 5. Conclusions and Future Perspectives

Since the discovery of MXene, it has been widely investigated for various applications; however, less work has been done on MXene-based membranes for water purification, desalination, and antibacterial applications [104,105]. In this review, we have summarized the recent progress of MXene-based membranes for water purification and antibacterial applications from both a theoretical and an experimental point of view. Different fabrication and modification methods for MXene laminates have been highlighted. The current challenge is the scalable production of MXenes because these preparation methods are expensive with a time-consuming synthesis, low yield, purity, and can be environmentally incompatible.

Most of existing studies were limited to a specific MXene, i.e., Ti_3_C_2_T_x_. Therefore, other members of same family need to be explored to fully utilize their power in separation applications. In addition, MXene-based membranes are only used for particular types of dye molecules and ions. The separation of small molecules (dyes or ions) that have a diameter less than 5 Å is still a big challenge for MXenes. Moreover, MXene-based membranes are still absent in other separation applications such as electrodialysis, organic separation nanofiltration, and forward osmosis. In term of selectivity, permeance, and separation applications, MXene is far behind graphene. Therefore, MXene needs to be explored for novel types of separation in future and to improve its permeance and selectivity.

The stability of MXene in water-based applications is another key issue; it can easily be degraded under humid conditions. MXene contains various functional groups responsible for good hydrophilicity such as GO membranes. Due to the hydrophilic properties of MXene, it can easily form hydrogen bonding with water molecules that results in degradation and delamination into water. This can possibly be modified by MXenes with a number of materials including polymers, nanoparticles, 2D sheets, and 3D materials. Efforts should be carried out in this direction to improve the stability of membranes by utilizing their functional groups with suitable crosslinking agents. Furthermore, focus should be given to controlled interlayer spacing, shape, size, and atomic defects within MXenes to improve the separation performance.

As for antibacterial studies, only Ti_3_C_2_T_x_ MXene has been tested against particular types of bacterial strain. More work is needed to use different type of MXenes and pathogens with different concentration. The bactericidal mechanism is still not clear. More theoretical and experimental investigations are required to fully understand the mechanism. Overall, when compared with graphene, MXene is still in an early stage with much more research needed, particularly on water purification, desalination, and antibacterial properties. 

## Figures and Tables

**Table 1:** MXene-based membranes for the separation of ions, molecules, and pathogens from water.

Type of Membrane	Fabrication Method	Feed Solution/Concentration	Rejection (%)	Permeability (Lm^−2^ h^−1^ bar^−1^)	Ref.
Ti_3_C_2_Tx	Vacuum filtration	RB	85	1084	[64]
		EB	90		
		CC (Each 10–20 mg/L)	97		
Ti_3_C_2_Tx	Vacuum filtration	NaCl (10,000 mg/L)	56–64	10	[100]
		BSA (2000 mg/L)			
Ti_3_C_2_Tx	Vacuum filtration	CR	92	405	[74]
		GN	80		
		MgCl2	2.3		
		Na2SO4	13.2		
		NaCl (Each 100–1000 mg/L)	13.8%		
Ti_3_C_2_Tx	Vacuum filtration	*E. coli*	>99	37.4	[91]
		*B. subtilis*	>99		
Ti_3_C_2_Tx	Vacuum filtration	Na_2_SO_4_	50–99	5–15.25	[101]
		Mg_2_SO_4_			
		MgCl_2_			
		NaCl			
		VOSO_4_			
Ti_3_C_2_Tx-Ag	Vacuum-assisted filtration	RB	79.9	~420	[84]
		MG	92.3		
		BSA (50–100 mg/L)	>99%		
Ti_3_C_2_Tx-GO	Vacuum filtration	BB	95.4	~25 L	[65]
		Rose Bengal	94.6		
		MLB	40		
		MLR	5		
		MgSO4	<1		
		NaCl (Each 10 mg/L)			
Ti_3_C_2_Tx-GO	Vacuum filtration	RB	>97 (dyes)	89.6	[102]
		MB			
		CV			
		NR (Each 10 mg/L)			
		Na_2_SO_4_	61		
		NaCl (Each 5 mM)	23		
Ti_3_C_2_Tx-GO	Vacuum filtration	Chrysoidine G	>99% (dyes)	71.9	[80]
		MLB			
		NR			
		CV			
		BB			
		HA			
		BSA			
		Na_2_SO_4_	61		
		NaCl (Each 10 mg/L)	23		
Ti_3_C_2_Tx-GO	Vacuum filtration	MO	>95	~8.5–11	[101]
		MLB			
		Acid yellow 14			
		IC			
		Eosin (Each 10 mg/L)			
Ti_3_C_2_Tx-TiO_2_	Spin coating	Dextran (3000 mg/L)	>95	~90	[103]

CC: cytochrome C; MLB: methylene blue; RB: rhodamine B; EB: Evan blue; MO: methyl orange; IC: indigo carmine; HA: humic acid; BB: brilliant blue; NR: neutral red; CV: crystal violet; CR: Congo red; GN: gentian violet; MG: methyl green; MLR: methylene red.
